# Impact of coronary bifurcation angle on the pathogenesis of atherosclerosis and clinical outcome of coronary bifurcation intervention–A scoping review

**DOI:** 10.1371/journal.pone.0273157

**Published:** 2022-08-17

**Authors:** Yoshinobu Murasato, Kyohei Meno, Takahiro Mori, Katsuhiko Tanenaka

**Affiliations:** Department of Cardiology, Clinical Research Institute, National Hospital Organization Kyushu Medical Center, Fukuoka, Japan; Osaka University Graduate School of Medicine, JAPAN

## Abstract

**Background:**

A coronary bifurcation stenting is still a challenging issue due to frequent restenosis and stent thrombosis even with drug-eluting stents. The bifurcation angle (BA) between a main vessel and a side branch is one of the crucial determinants of coronary flow and shear stress that affect the plaque distribution. Previous bench and clinical studies have evaluated the impact of the BA between the proximal main vessel and the side branch (Angle A) and the BA between the distal main vessel and the side branch (Angle B) on the clinical outcomes of bifurcation stenting. However, the impact has not yet been fully elucidated due to a lack of statistical power or different manner of the assessment of BA.

**Objectives:**

To analyze the published studies on coronary artery BA, the modalities used for assessment, and the impact of BA on interventions and attempt to define the pre-procedural protocols.

**Data sources:**

A scoping review was performed using the Joanna Briggs Institute Methodology. A total of 52 relevant references were selected from PubMed, Cochrane Library, and CINAHL databases and categorized into three topic areas.

**Results and conclusions:**

A wider Angle A is associated with the increased likelihood of carina shift and a wider Angle B, with that of side branch occlusion. A wider Angle B promotes stent malapposition and deformation in the side branch ostium and has been reported as an independent predictor of major adverse cardiac events after bifurcation stenting; however, improvement of the drug-eluting stent, refinement of the stenting technique, and accurate 3-dimensional assessment may attenuate the adverse clinical impact of a wider BA.

**Implications of key findings:**

Assessment of the BA is necessary to predict the effect of bifurcation intervention procedure on the stent configuration and coronary flow at the bifurcated vessels. This will help to optimize stent selection and the stenting technique.

## Introduction

Percutaneous coronary intervention (PCI) of bifurcation lesions accounts for 15–20% of all cases [1] and is still a challenging issue due to frequent restenosis and stent thrombosis.[1,2] More frequent stent malapposition [3–8], subclinical thrombus attachment [3], flow disturbance [9,10], increasing the area of low wall shear stress [9], and inappropriate vessel dilation which is against the vascular branching law [11] are possible explanations for worse clinical outcomes in bifurcation PCI. The bifurcation angle (BA) not only determines the nature and pathology of the bifurcation lesions but is also strongly associated with restenosis or stent thrombosis.

In this scoping review, the nature of the coronary bifurcation and its clinical impact on bifurcation PCI, as reported in the published studies, is comprehensively described, and analyzed.

## Methods

**Fig 1:**
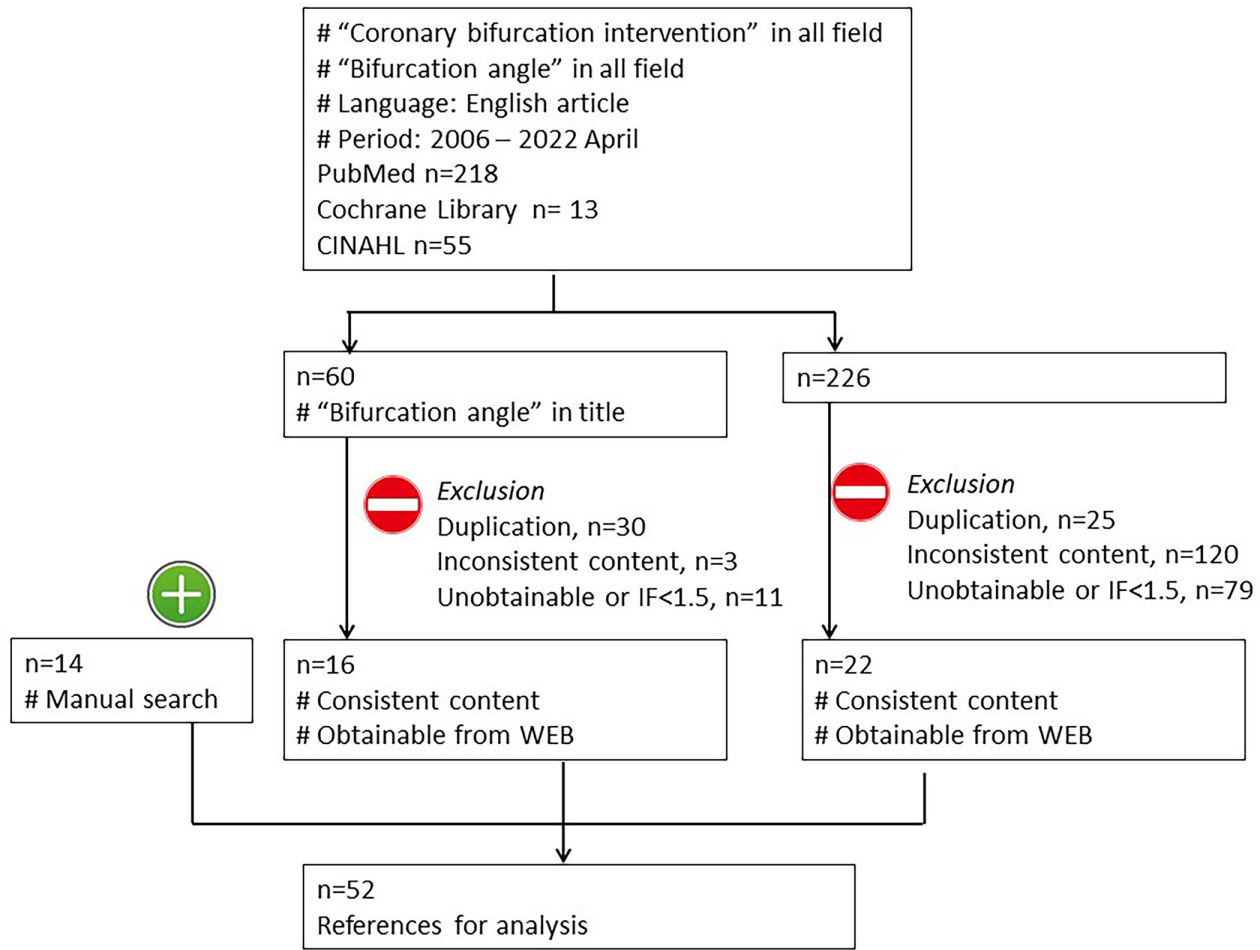
Flow chart of search strategy. IF: Journal impact factor.

A comprehensive search was performed in three medical online databases (PubMed, Cochrane Library, and Cumulative Index to Nursing and Allied Health Literature [CINAHL]) (Fig 1). Papers, published in English between 2006 and the present (2022), containing the terms “coronary bifurcation” and “bifurcation angle” either in the title, abstract or text of the paper were extracted (286 records). Next, the papers in which the title included “bifurcation angle” were searched (60 records) and the following papers were excluded: (1) duplicated papers (30 records), (2) papers in low impact journals (<1.5) or unable to be downloaded from the WEB system (11 records), and (3) papers where the content was inconsistent with the topic of this review (3 records). The papers that did not contain the term “bifurcation angle” in the title (226 records) were also searched in the same manner with the following exclusion: (1) 25 records, (2) 79 records, and (3) 120 records. Bibliographies related to the topics were manually searched and reviewed, and relevant studies were then included (14 records). Finally, 52 records were selected and categorized into three topic areas for the scoping review: (1) Definition of BA and analysis methods; (2) Effect of BA on bifurcation geometry, physiology, and pathology; (3) Impact of BA on the clinical outcome of bifurcation stenting. The selection of papers was performed independently by four reviewers and based upon the population, concept, and context guidelines specified in the Joanna Briggs Institute Methodology for JBI Scoping Reviews [12]. The review had not been registered.

## Review and discussion

### 1. Definition of BA and analysis methods

#### 1.1 Two-dimensional quantitative coronary angiography (2D QCA)

**Fig 2:**
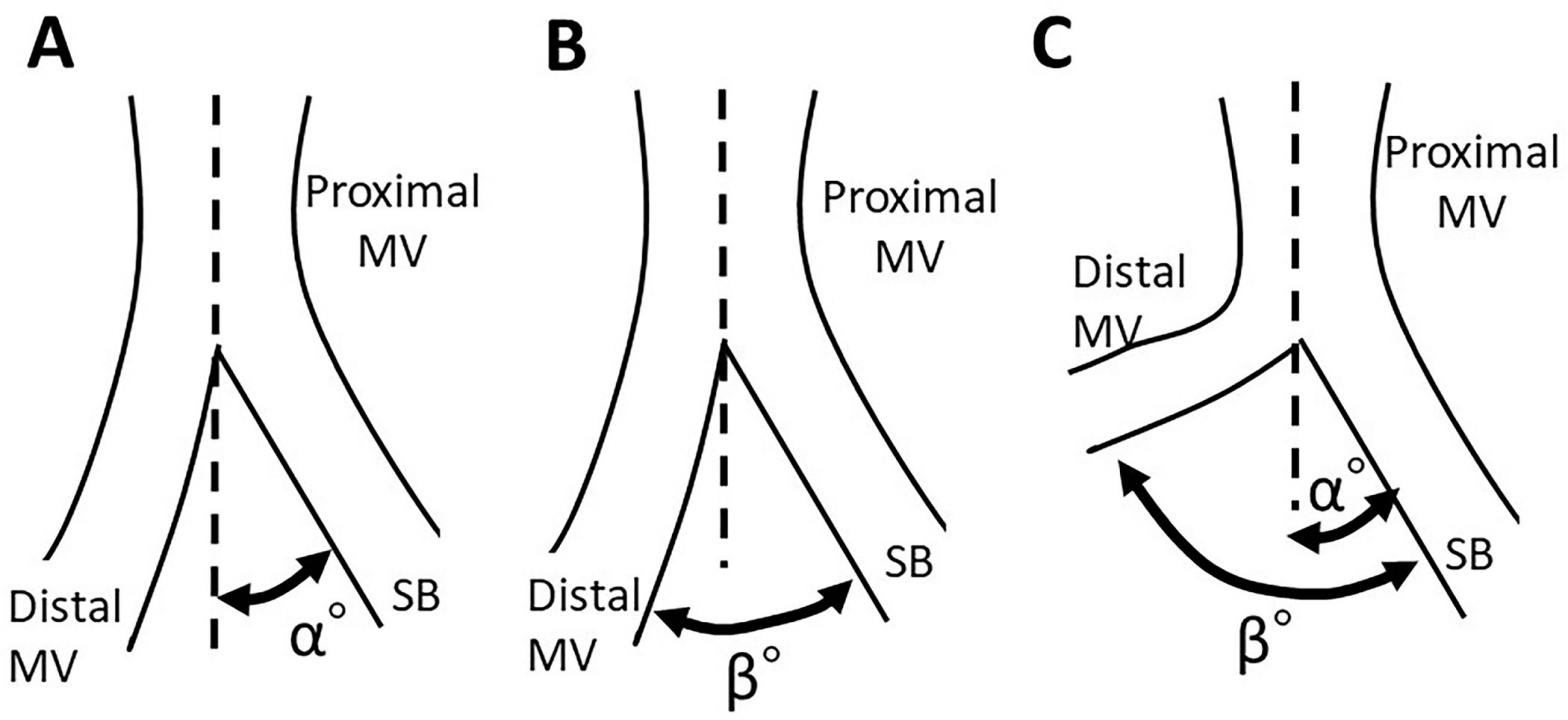
Definition of bifurcation angle (BA). A: Definition in in the Bifurcation Academic Research Consortium. Angle A: BA between proximal main vessel (MV) and side branch (SB), Angle B: BA between distal MV and SB, Angle C: BA between proximal and distal MV. B: Another assessment of BA related to MV and SB. BA-α: Angle between axis of proximal MV and SB. C: Discordance of BA assessments. In the case with large curvature on the MV side, the Angle B is assessed as a wide BA in which the angle change due to the curvature is included, although the BA-α is not affected by the curvature.

Recently, the definition of BA has been standardized in the Bifurcation Academic Research Consortium as shown in Fig 2A: the BA between the proximal main vessel (MV) and the side branch (SB) is defined as Angle A, the BA between the distal MV and the SB is defined as Angle B, and the BA between the proximal and the distal MVs is defined as Angle C. The BA is measured in the optimal projection with clear visualization of branches in the maximal BA without any vessel overlap or foreshortening [13].

However, two modes of defining the BA related to the MV and SB have been reported to date, which has led to some confusion in the understanding of the results of each analysis. Although the angle between the centerline of the proximal MV and the SB (BA-α, complementary angle of Angle A) has been defined as the BA in several reports [14–20] (Fig 2B), Angle B has been considered as the more accepted form of the BA [21–29]. The curvature of the vessels is responsible for the difference in the assessment of the BA by these two methods. If the vessels have a larger curvature on the MV side, the latter method will give a wider BA (Angle B) since it includes the vessel curvature; however, the former method is not affected by this curvature, which is regarded as narrower BA-α (Fig 2C).

#### 1.2 Three-dimensional (3D) quantitative coronary angiography (QCA)

In 2D QCA, it is difficult to find an optimal projection avoiding vessel overlap and foreshortening in every case due to the complex anatomy of the bifurcation lesions. 3D QCA, in which the 3D coronary tree is reconstructed by using two orthogonal images, provides a more accurate, sensitive, and reproducible BA measurement in an appropriate projection in a 3D phantom model [30]. In a sub-study of the SYNTAX trial, the average diastolic left main coronary artery (LMCA)-left anterior descending artery (LAD) [Angle C] and LAD-Left circumflex (LCX) angles [Angle B] were reported to be 105.9 ± 21.7° and 95.6 ± 23.6°, respectively [31].

#### 1.3 Cardiac computed tomography angiography (CCTA) (Table 1)

CCTA provides additional 3D information on the bifurcated area and accurate BA measurement in an optimal view [32–35]. According to the CCTA studies which included >200 patients without significant coronary stenosis [32,34], the average LMCA BAs (Angle A: LMCA–LCX, Angle B: LAD–LCX, Angle C: LMCA–LAD) were 126–143°, 72–79°, and 121–139°, respectively [32,34,35]. This reported Angle B was smaller than that found by 3DQCA in the sub-study of the SYNTAX trial [31] and the difference is derived from more significant LMCA lesions in the SYNTAX study cohort. A wider Angle B (>70°) was more dominant in LMCA bifurcation (26%) compared to other bifurcations [32].

**Table 1:** Previous reports concerning coronary bifurcation angle on cardiac computed tomography angiography.

Bifurcation	Studies	Medrano-Gracia [34]		Kawasaki [32]		Ellwein [35]		Juan [43]				Cui [44]			
	Subject	No CAD	N	CAD suspect	n	CAD suspect	n	LMCA disease (-)	n	LMCA disease (+)	n	LMCA stenosis <50%	n	LMCA stenosis >70%	n
LMCA	Angle A	126.1±21.1	300	143±13	209	147.6 [144.3,151.0]	67	ND		ND		145.2 ± 14.7	37	145.5 ± 13.2	45
	Angle B	78.9±23.1		72±22		ND		75.53 ± 14.71	40	87.34 ± 18.84*	211	68.3 ± 18.0		80.0±19.2*	
	Angle C	138.6±18.9		121±21		ND		ND		ND		140.5 ± 27.0		137.1 ± 20.6	
Diagonal	Angle A	145.1±14.8	242	ND		161.1 [158.3,163.9]	67	ND		ND		ND		ND	
	Angle B	51.9±16.4		ND		68.7 [64.1, 72.6]		ND		ND		ND		ND	
	Angle C	150.5±13.8		138±19	209	ND		ND		ND		ND		ND	
OM	Angle A	146.9±21.5	176	ND		148.0 [142.7,153.3]	67	ND		ND		ND		ND	
	Angle B	55.8±23.2		ND		71.4 [66.4, 76.3]		ND		ND		ND		ND	
	Angle C	145.0±15.7		134±23	209	ND		ND		ND		ND		ND	
RCA	Angle A	ND		152±15	209	ND		ND		ND		ND		ND	
	Angle B	ND		137±20		ND		ND		ND		ND		ND	
	Angle C	ND		61±21		ND		ND		ND		ND		ND	

LMCA: left main coronary artery, OM: Oblique marginal branch, RCA: Right coronary artery, CAD: Coronary artery disease, ND: Not described, Values are expressed as mean ± standard deviation or median [1^st^, 3^rd^ quartile]

* p<0.05 in the comparison between the groups divided by the presence of LMCA stenosis.

#### 1.4 Optical coherence tomography (OCT)

Longitudinal OCT observation enables validation of the BA measurement against CCTA [36]. Watanabe et al. [36] investigated the angle of the carina tip as well as the Angle B between the distal MV and SB and found that these angles were not always consistent. Some cases had long carinal tips with narrower angles but had a wider Angle B.

### 2. Effect of BA on bifurcation geometry, physiology, and pathology

#### 2.1 SB ostial length, area, and shape

**Fig 3:**
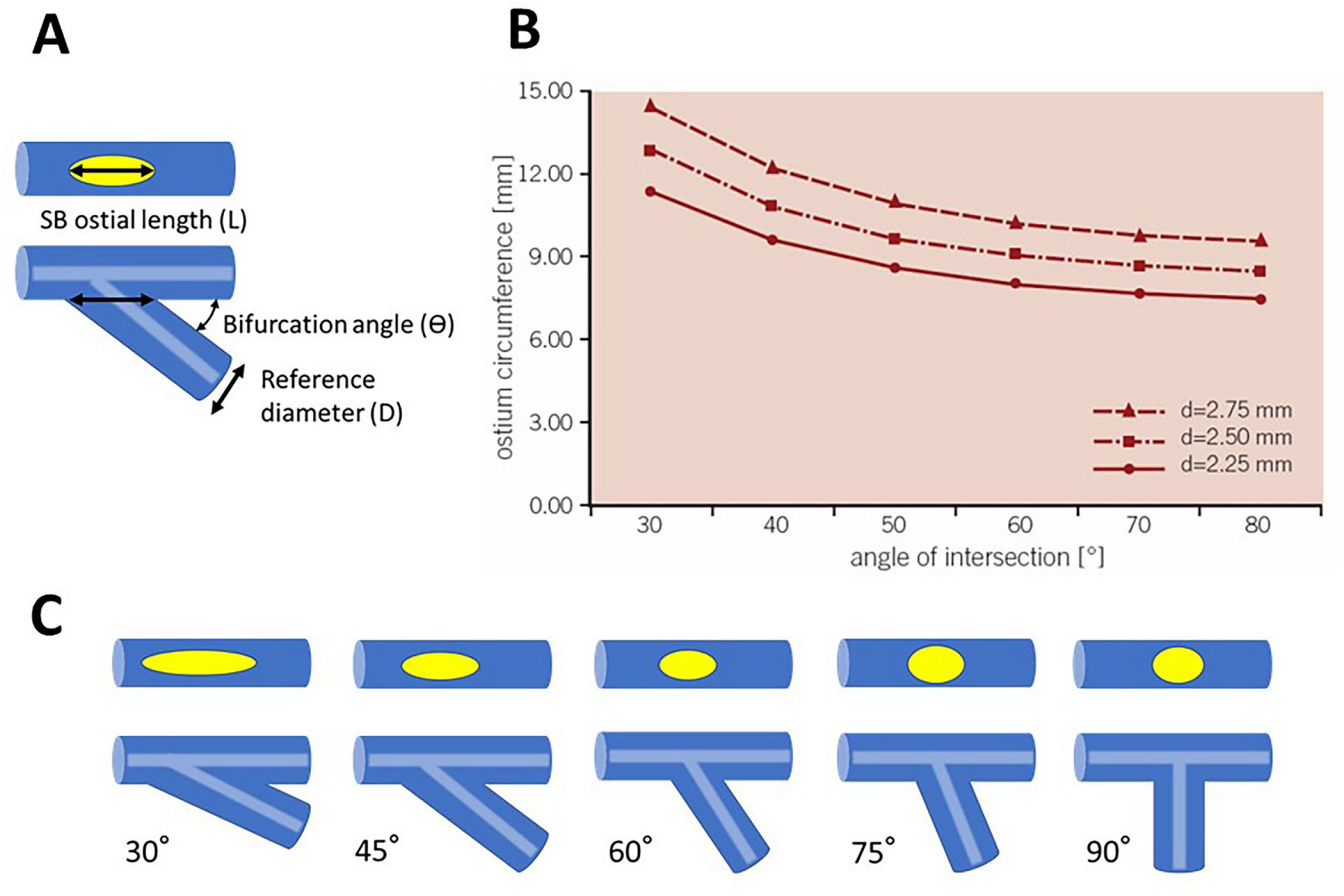
Relation between bifurcation angle (BA) and side branch (SB) ostial geometry. (A) Relation among BA (θ), SB ostial length (L) and reference diameter (D). See detail in text. (B) SB ostial circumferential length. Narrower BA is associated with longer length. Adapted from reference no. 36. (C) SB ostial shape. Narrower BA is associated with elliptical change in SB ostial shape.

The BA has a definite effect on the side branch (SB) ostial geometry. The SB ostial length is theoretically calculated using the following formula,

$$L=\frac{D}{\sin\;\theta }$$

where L, D, and θ are ostial length, reference diameter of the SB and BA between distal MV and SB, respectively (Fig 3A). Accordingly, the narrower angled bifurcation has a longer SB ostial length and a more elliptical shape (Fig 3B, 3C) [37,38]. The bifurcation with a narrow BA requires wider SB ostial dilation than estimated from the distal reference, which is likely to result in a geographic miss and inadequate expansion of the SB ostium.

#### 2.2 Shear stress

A bifurcated vessel divides the coronary flow into daughter vessels and generates a coexistence of low wall shear stress on the lateral side and high shear stress on the carinal side of the vessels. The resulting turbulent flow on the lateral side is likely to promote atherosclerosis, whereas the high flow prevents this on the carinal side [9]. The magnitude of reversed flow and the extension of the recirculation zone are more prominent in a wide BA [39]. A wider BA (Angle B) leads to the dispersal of high wall shear stress and its gradient impact zone on the adjacent arterial wall, which has been shown to lead to aneurysm formation in the middle cerebral artery [40].

#### 2.3 Plaque distribution

Yakushiji et al. compared the LAD / diagonal branch (D) bifurcation with Angle B of 51.5±19.5° (n = 58) and LMCA bifurcation with Angle B of 97.7±19.1° (n = 81) in the intravascular ultrasound analysis and demonstrated that plaque distribution was similar in the two bifurcations [41]. Continuous plaque extension from proximal to distal MV was observed in >90% of cases in both bifurcations. The SB had less calcium and a smaller plaque burden compared to the proximal or distal MV [41].

Higher levels of untreated low-density lipoprotein cholesterol were associated with the presence of a bifurcation lesion with a wide Angle B >80° (odds ratio 3.77, 95% CI 1.05–13.5) [42].

#### 2.4 Lesion distribution

A predominance of wider Angle B between LAD and LCX in the CCTA was found in the group with LMCA bifurcation disease compared to the group without it [43–45]. The lesions with wider Angle B in the LMCA were more likely to be present in male patients and those with a higher body mass index (risk ratio; 2.07 and 2.54, respectively) [45]. The presence of high-risk (71±19° vs. 55±19°) and non-calcified plaques (74±20° vs. 50±14°) in the proximal MV was associated with wider Angle B values [46]. A narrower Angle A in the LMCA in patients with chronic kidney disease was likely to present with more calcified lesions [20]. Another CCTA study revealed that angle change due to the cardiac motion was more prominent in the Angle A (LMCA-LCX angle) compared to the Angle C (LMCA-LAD angle) [33].

### 3. Impact of BA on clinical outcome of bifurcation stenting

#### 3.1 Two-stenting technique

**Fig 4:**
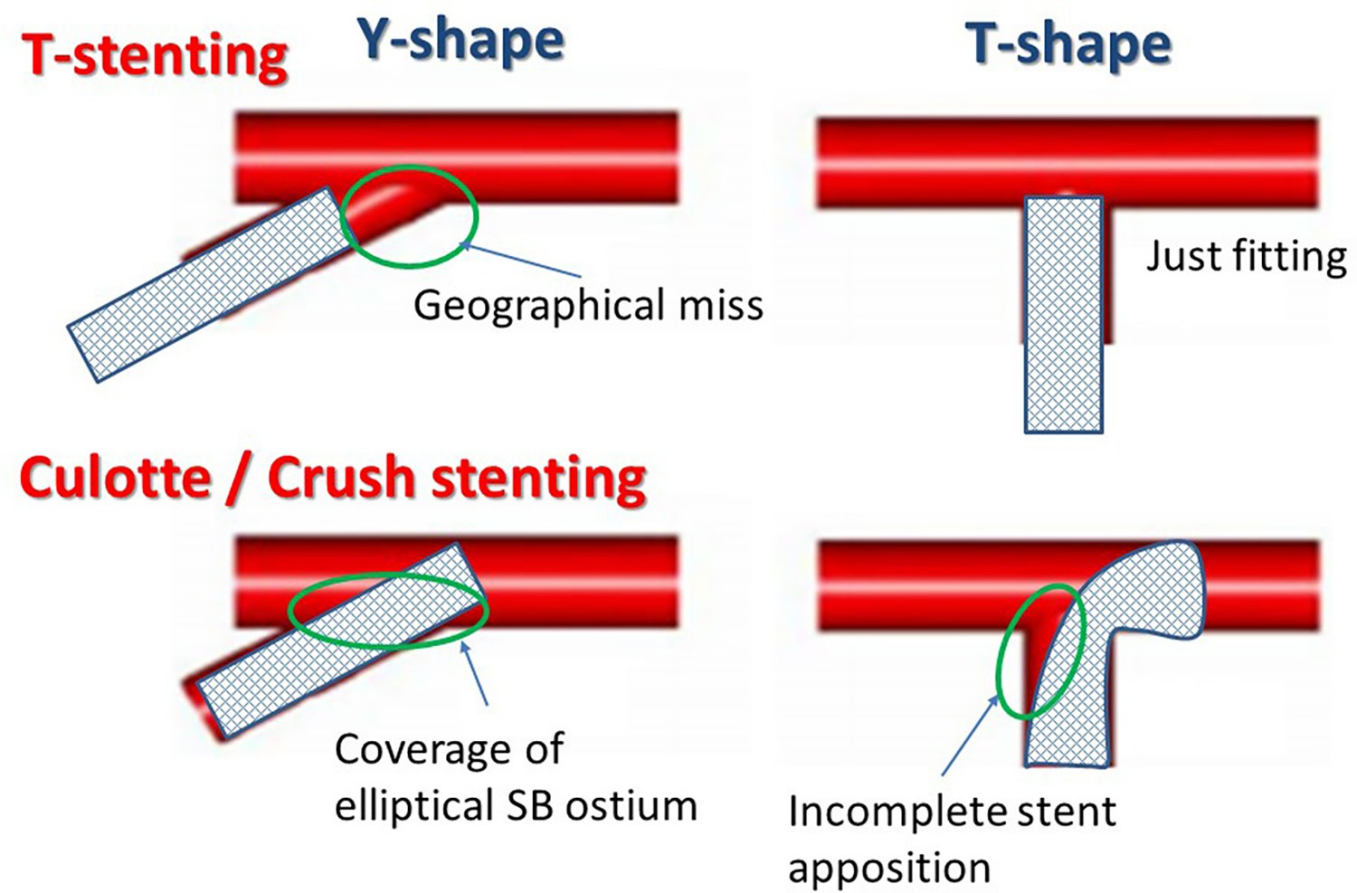
Favorable stenting technique in Y- and T- shape bifurcation lesions. In the Y-shape bifurcation, culotte or crush stenting is favorable due to complete coverage of the extended SB ostium. In the T-shape bifurcation, stenting crossing from the proximal MV to the SB is not suitable due to more frequent stent malapposition in the carinal area. See detail in the text.

As per the European Bifurcation Club consensus, assessment of the BA, namely distinguishing the Y- or T- shapes, is recommended to determine the optimal two-stenting technique [47] (Fig 4).

The obliquely extended ostium in the Y-shape bifurcation with narrower angled SB promotes geographical miss or protrusion of the SB stent into the MV when it is implanted in the SB ostium. T-stenting is not suitable in the Y-shape bifurcation, because the protrusion is crushed during MV stenting. Culotte or crush stentings are more suitable in the Y-shape bifurcation due to definite coverage of the extended SB ostium area and fewer chances of a geographical miss. On the contrary, in the T-shape bifurcation, T-stenting is ideal when the SB stent is appropriately implanted to cover the SB ostium without any geographic miss. The right-angled bifurcation is prone to having incomplete coverage of the SB carinal site due to inadequate stent expansion in the SB ostium. An observational study of OCT in bifurcation stenting indicated that malapposed strut-vessel wall distance in the MV was apt to be larger in the SB ostial level in two-stent deployment compared to simple MV stenting [4].

**Fig 5:**
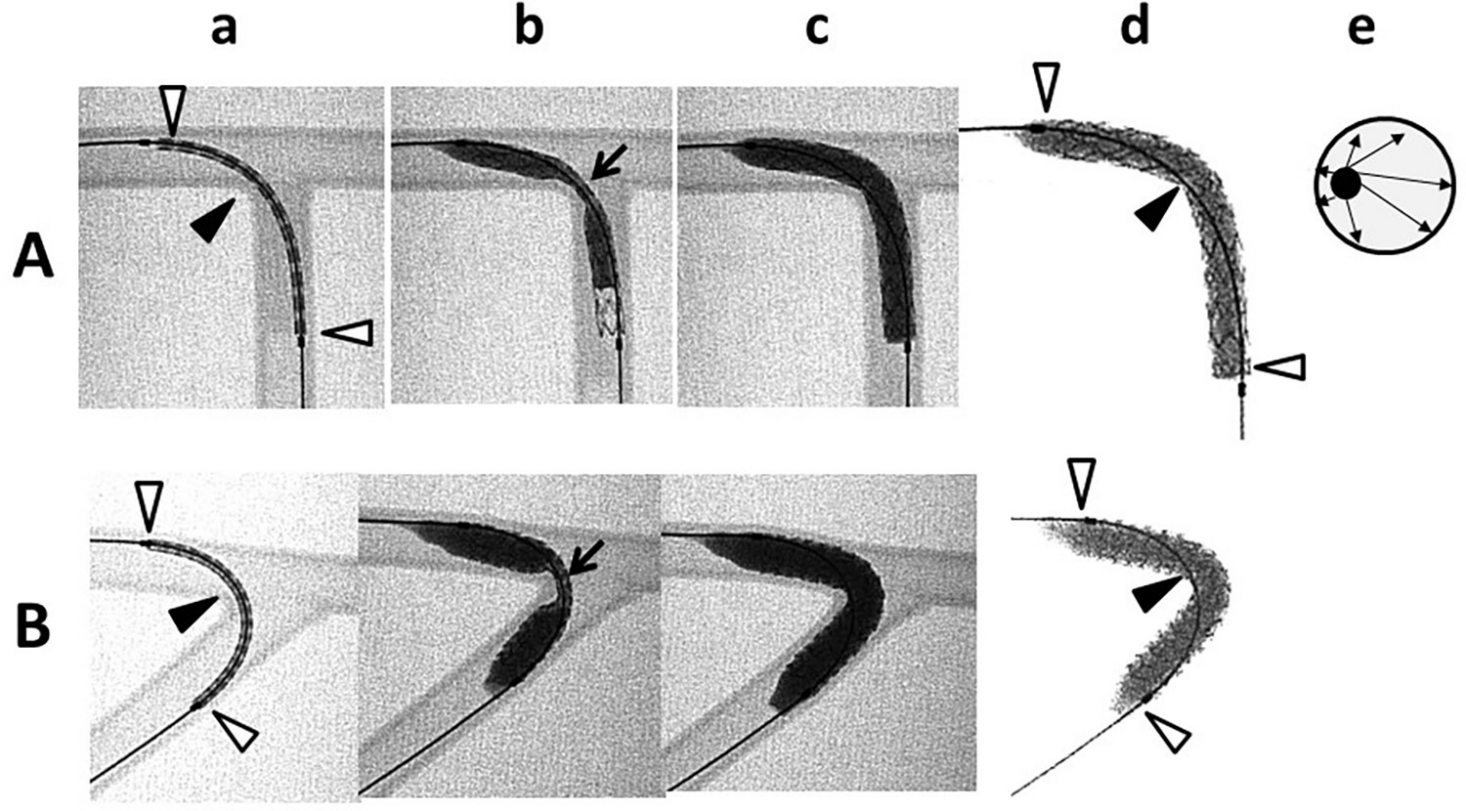
Experiment of the SB stenting from the MV. (A) Right-angled bifurcation. (B) Steeply angulated bifurcation at 45° angle between the proximal MV and the SB. (a) Initial position of the guide wire is on the inner side at the middle portion (black triangle) and towards the outer sides at both ends of the stents (white triangles). (b) In the initial phase of the inflation, the middle portion was dilated at the end (arrow). The stent was thus dumbbell shaped. (c) There was unstented area at the distal carina even after full expansion of the stents. (d) The guide wire position had not been changed during the stent expansion. Note the biased position of the wire which was in the central core in the balloon. (e) Scheme of cross section of the stent balloon inflation with the deviated position of the guide wire (black circle) at the SB ostium. Adapted from Fig 17 of reference no. 7.

**Fig 6:**
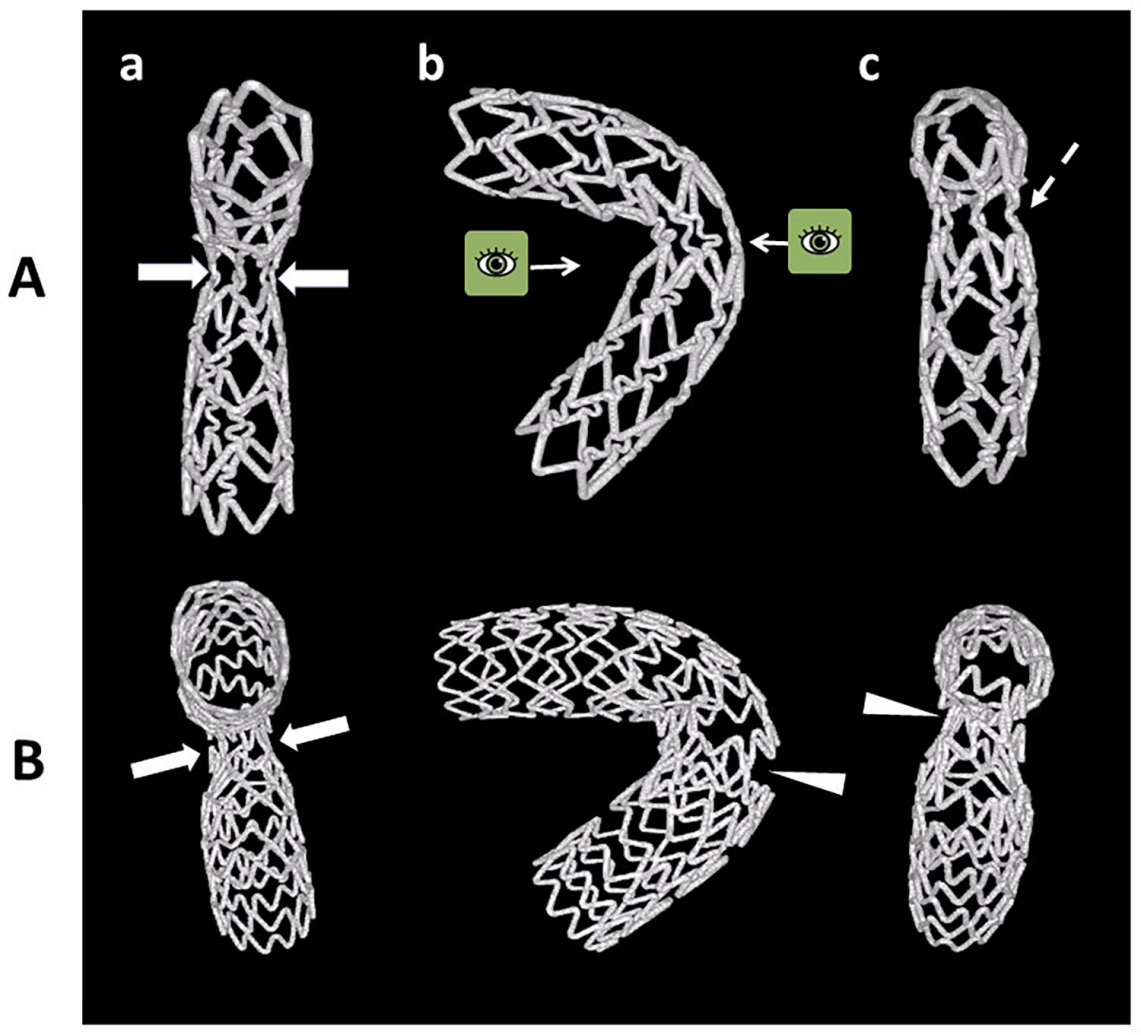
Micro-focus computed tomography images of the SB stents deployed from the MV (a: View from the inner side, b: Anterior—posterior view, c: View from the outer side). Experiments were performed using tube-slotted type stent (A) and coil type stent (B). There were restrictions of the stent expansions in both stents (A[a], B[a], arrows). The outer struts of the tube-slotted type stent were stretched (A[c], dotted arrow), whereas the coil type stent had wide opening between the coils at its outer side (B[b], [c], white triangles). Adapted from reference 7, Fig 18.

In the bench testing [5–8], Angle B is also a crucial factor for the SB stent expansion in the LMCA model, which is likely to have a wider Angle B, such as right and extremely steep angles shown in Fig 5A and 5B. Initially the stent is inflated in the proximal and distal sites (Fig 5A–5B) and, finally, the middle part, located in the SB ostium, is expanded (Fig 5A–5C). However, the guidewire position is maintained during stent expansion, which is located on the outer side of the stent in both proximal and distal sites (Fig 5A—a and d, white arrows) and on the inner side of the stent at the SB ostium (Fig 5A- a and d, black arrows). Therefore, the stent expansion at the SB ostium is limited due to the deviated position of the guidewire in the central port of the stent balloon on the inner side of the curvature (Fig 5A-e), and stretched struts or widely opened intra-cellular space are found on the outer side (Fig 6).

In the bench study replicating a 3-dimensional LMCA bifurcation model, there was a positive correlation between the Angle A (LMCA–LCX angle) and the expansion ratio of the LCX stent in crush stenting [5]. Narrower Angle A and wider Angle B restricted stent apposition at the SB ostium [5]. A clinical study investigating the effect of bifurcation stenting on BA change demonstrated that a two-stent technique decreased Angle B by 10° after the procedure and the BA change was more evident in a wide- Angle B (>70°) and in Crush stenting. [48] Another clinical study of LMCA stenting also revealed a significant decrease of Angle B from 82 ± 27° to 72 ± 28°. [49] In addition, a more dynamic hinge motion in wider Angle B increases the possibility of SB stent fracture which may lead to restenosis [7,50]. Although final kissing balloon inflation is necessary for removal of the SB-jailing struts and sufficient stent expansion for the two-stent technique, a narrower Angle B [51] and wider Angle C [52] are predictive of failure of the procedure. Under expansion of the stent at the bifurcation or bigger stent size selection, which goes against contemporary vascular branching law, promotes the lowering of wall shear stress and an increase in its gradient and oscillation [10,11,53].

#### 3.2 Impact on the clinical outcome (Table 2)

Since the BA affects stent expansion, stent conformation, the durability of the polymer according to the curvature, and access to the SB, these abilities are hampered due to a wider BA which may have an adverse effect on the clinical outcome of bifurcation PCI [14,21]. However, improvement of drug-eluting stent technology, refinement of the stenting technique, and imaging-guided PCI [54] have helped in a reduction in major adverse cardiac events (MACE) and the attenuation of adverse effects of wider BA [1]. Therefore, there are controversial reports currently on the impact of the BA on clinical outcomes after bifurcation PCI.

**Table 2:** Previous reports concerning impact of bifurcation angle on mid- to long-term clinical outcome after coronary bifurcation stenting.

		Sample size	Angle measurement	Stenting treatment	Follow-up	Main finding
***Significant impact***						
	Dzavik et al. [14]	133	2D QCA	2-stent	12 months	The high BA-α (low Angle A) group had worse MACE rate than the low BA-α (high Angle A) group (22.7% vs 6.2%).
	Collins et al. [15]	140	2D QCA	2-stent (crush / culotte)	27 months	low BA-α (high Angle A)was associated with better MACE-free survival rate (OR = 0.59, 95% CI 0.35–0.92) than the high BA-α (low Angle A).
	Freixa et al. [16]	360	2D QCA	2-stent (crush 304/ culotte 56)	49 months	BA-α <50°(Angle A >130°) was associated with a lower risk of MACE or CCS Class ≥ 2 angina (OR = 0.59, 95% CI 0.35–0.92)
	Adriaenssens et al. [21]	134	2D QCA	2-stent (culotte)	9 months	The Angle B over means of 52° was related to more frequent restenosis compared to lower Angle B with < 52° (24.1% vs. 19.6%).
	Chang et al. [25]	238	2D QCA	2-stent (culotte)	38 months	Higher Angle B was an independent predictor for target lesion failure (OR 3.484, CI 1.213–10.009)
	DK-CRUSH III [26]	419	2D QCA	2-stent (DK-crush 210 / culotte 209)	12 months	Higher Angle B (>70°) was associated with more frequent 1-year MACE in culotte stenting than in DK-crush stenting (OR 0.20, CI 0.08–0.49).
	COBIS [55]	462	2D QCA	2-stent in LMCA	35 months	Higher Angle C was an independent predictor of target lesion failure after crush stenting with the best cut-off value of 152°, while it was not after T-stenting.
	J-CYPHER [22]	945	2D QCA	2-stent vs. 1-stent in LMCA	12 months	Angiographic SB restenosis after 2-stent was more frequent compared to 1-stent (35.3% vs. 14.5%) in the LMCA bifurcation with higher prevalence of Angle B >70° (40%).
	NORDIC I & BBC-ONE [29]	913	2D QCA	Provisional vs. Complex stenting	9 months	Higher Angle B (>60–70°) brought more MACE in complex stenting (simple 9.6% vs. complex 15.7%).
	Amemiya et al. [56]	170	3D QCA	1-stent in LMCA	12 months	Lower Angle C was associated with higher MACE rate (low 33.3%, middle 14.3%, high 8.9%).
	Konishi et al. [19]	177	CCTA	LMCA stenting	11 months	Patients with intra-stent restenosis presented smaller Angle A than those without the restenosis (52.2± 14.5° vs. 32.0± 18.1°).
	MITO [57]	300	3D QCA	2-stent in LMCA	36 months	Large Angle B change (>7.2°) between systolic and diastolic phase was associated with higher TLF rate compared to the small BA change. (Adjusted HR 5.85; 95%CI, 3.40–10.1).
	Watanabe et al. [27]	55	OCT	1-stent	12 months	Angle B was negatively corelated with uncovered strut percentage and positively with neointimal thickness in the SB ostial region.
***Insignificant impact***						
	Collins et al. [15]	266	2D QCA	1-stent	27 months	No effect of BA on clinical outcome
	DK-CRUSH I [23]	220	2D QCA	2-stent (DK-crush)	8 months	No significant difference in the MACE between the low (<60°) and high Angle B (≥60°) in the lesion (low Angle B 17.61% vs. high Angle B 17.64%).
	COBIS [28]	1,432	2D QCA	All bifurcation stenting	21 months	Higher Angle B (≥50°) group presented a similar MACE rate as lower Angle B (<50°) group (6.6 vs. 6.9%)
	SYNTAX [31]	226	3D QCA	LMCA stenting	12 months	MACCE rate were 17.2%, 14.6%, and 18.9%, respectively in the three groups of <82°, 82°-106°, and >107° of diastolic Angle B (non-significant)
	SYNTAX [58]	185	3D QCA	LMCA stenting	60 months	MACCE rate increased to 37.1%, 37.7%, and 35.6%, respectively in the three groups of <82°, 82°-106°, and >107° of diastolic Angle B (non-significant)

QCA: Quantitative coronary angiography, CCTA: Cardiac computed tomography angiography, OCT: Optical coherence tomography, LMCA: Left main coronary artery.

BA: Bifurcation angle, MACE: Major adverse cardiac events, OR: Odds ratio, CI: Confidential interval, CCS: Canadian Cardiovascular Society functional classification.

LAD: Left anterior descending artery, MACCE: Major adverse cardiac and cerebrovascular adverse events.

*3*.*2*.*1 The significant impact of BA*. Dzavík et al. [14] investigated the impact of the BA-α, which was the complementary angle of Angle A, on the clinical outcome of crush stenting in 133 bifurcation lesions. They compared wide and narrow BA-α groups divided by the median value (50°, Angle A 130°) and found that the wide BA-α (narrow Angle A) group had a worse 12-month MACE rate than the narrow BA-α (wide Angle A) group (22.7% vs 6.2%). In their prospective bifurcation registry [15], after a median follow-up of 26.5 months, the narrow BA-α (wide Angle A) was associated with better MACE-free survival rate (OR = 0.59, 95% CI 0.35–0.92) compared with the wide BA-α (narrow Angle A) in 140 lesions treated with crush/culotte stenting, but it did not affect the outcome of 266 lesions treated with single MV stenting. After a median follow-up of 4.1 years, BA-α <50° (Angle A >130°) was associated with a lower risk of MACE or CCS Class ≥ 2 angina (OR = 0.59, 95% CI 0.35–0.92) in those treated with either crush (n = 304) or culotte stenting (n = 56) [18].

Adriaenssens et al. [21] reported that a wider Angle B was an independent predictor for angiographic restenosis 6–8 months after culotte stenting in 134 bifurcation lesions. The Angle B above the mean value of 52° was related to more frequent restenosis compared to that below 52° (24.1% vs. 19.6%). Chang et al. also reported that a wider Angle B was an independent predictor for target lesion failure (TLF, OR 3.484, 95%CI 1.213–10.009) after an average follow-up of 3.2 years in 238 patients treated with culotte stenting in their retrospective study [25]. In the randomized comparison between double kissing (DK)-crush and culotte stenting in the DK-CRUSH III study, a wider Angle B (>70°) was associated with more frequent 1-year MACE in culotte stenting than in DK-crush stenting (OR 0.20, 95%CI 0.08–0.49) [26].

Ki et al. investigated the impact of the BA on clinical outcomes in 462 patients treated with LMCA two-stenting in the COBIS registry [51]. A wider LMCA-LAD angle (Angle C) was an independent predictor of TLF after crush stenting with a best cut-off value of 152°, but it was not in the cases after T-stenting [55]. A greater area with low shear stress on a long metal overlapping site might be responsible for more restenosis or thrombogenic events in the group with the wider Angle C.

Toyofuku et al. [22] investigated the angiographic outcome of the bifurcation stenting in the J-Cypher registry (LMCA, 945 lesions, and LAD–Diagonal, 1271 lesions). They demonstrated that angiographic binary restenosis in the SB after two-stent deployment was more frequent compared to one-stent deployment in the LMCA bifurcation (35.3% vs. 14.5%), where a higher prevalence of Angle B >70° was presented (40.1–44.7%). On the contrary, the SB binary restenosis after two-stent deployment was numerically less than one-stent deployment in the LAD–Diagonal bifurcation (15.1% vs. 22.6%) with lesser frequency of Angle B >70° (6.0–8.0%).

Amemiya et al. [56] investigated the impact of the BA on the MACE in 170 patients who underwent cross-over stenting in the LMCA bifurcation using 3-D QCA. The patients were divided into three groups based on Angle C (low: <128.0°, middle: 128.0–151.6°, high: >151.6°) and revealed that a low Angle C was associated with a higher 12-month MACE rate (low 33.3%, middle 14.3%, high 8.9%). The Angle C was identified as one of the independent predictors of MACE as well as stent diameter and bare-metal stent.

Konoshi et al. reported that patients with intra-stent restenosis after stent implantation in proximal LAD lesions presented with a narrower Angle A in the LMCA bifurcation than those without the restenosis (32.0° ± 18.1° vs. 52.2° ± 14.5°) [19].

Large Angle B changes (>7.2°) between the systolic and diastolic phase were also associated with a higher TLF rate at 3-year follow-up after LMCA two-stenting compared to the small Angle B changes in the MITO registry (n = 300, adjusted hazard ratio [HR] 5.85; 95%CI, 3.40–10.1) [57].

In the OCT study, the Angle B negatively correlated with the uncovered strut percentage and positively with neointimal thickness in the SB ostial region [27]. It suggested that a wide BA with flow stagnation behind the SB-jailing strut may promote neointimal hyperplasia.

*3*.*2*.*2 Non-significant impact of the BA*. Chen et al. [23] compared the 8-month MACE between wide (≥60°) and narrow (<60°) Angle B groups in 220 bifurcation lesions in the DKCRUSH-1 study and found no significant difference in the MACE in the lesions treated with DK-crush stenting (17.64% vs. 17.61%). They concluded that adequate stent apposition in the SB ostium was obtained after two-time kissing balloon inflation, which prevented worse outcomes in a wider BA bifurcation.

In the COBIS registry [28], the clinical impact of the BA was investigated in 1,432 patients with bifurcation lesions with a median follow-up duration of 21 months. The wider Angle B (≥50°) group presented a similar MACE rate as the narrow Angle B (<50°) group (6.6 vs. 6.9%) despite less utilization of the jailed wire technique and final kissing balloon inflation.

A sub-study of the SYNTAX trial in 226 patients eligible for 3-D QCA also failed to show any significant impact of Angle B in terms of major adverse cardiac and cerebrovascular events (MACCE) [31,58]. One-year MACCE rates were 17.2%, 14.6%, and 18.9%, respectively in the three groups of <82°, 82°-106°, and >107° of diastolic Angle B [31]. Five-year MACCE rates increased to 37.1%, 37.7%, and 35.6%, respectively, however, no significant difference was noted amongst the groups [58]. Although the analysis of diastolic Angle B restricted to the LMCA bifurcation (n = 185) also failed to show any difference in the MACCE, post-PCI systolic-diastolic range <10° was an independent predictor of the MACCE (hazard ratio [HR] 2.65). The diminished post-PCI systolic-diastolic range after complex bifurcation stenting compared to that with simple MV stenting (11.7 ± 7.8° vs. 9.8 ± 7.6°) implies that the unnatural shape of the bifurcation was corrected by the metal stent, and this prevented worse MACCE.

The impact of the BA on the clinical outcome after single-stenting was reported to be insignificant [15,29] or limited in LMCA bifurcation with steep SB curvature (Angle A <50°) [19] or MV curvature (Angle C<128°) [56]. Since long-balloon dilation in the steep-angled lesion is likely to cause stent deformation and increase malapposition [10,59,60], post dilation with a short balloon in the proximal MV (proximal optimization technique) [61], short overlapping kissing balloon inflation [10,62], and SB dilation with an ultra-short balloon [63] or proximal balloon edge [60] are effective to minimize bifurcation stent failure. These large-scale bifurcation PCI registry studies did not show a significant impact of the BA, given that they included a single-stent strategy in more than 70% of cases; therefore, the impact of the BA was less [28,31,58]. Most of the dedicated studies for two-stenting demonstrated that wide Angle B (>60–70°) resulted in worse clinical outcomes [21,25,26,29]. Steep SB curvature (Angle A <130°, BA-α>50°) was also associated with worse clinical outcomes [14–16]. Selection of the appropriate two-stenting technique according to the bifurcation anatomy (Y-shape: culotte or crush stenting, T-shape: T-stenting favorable) is important. In case of the extreme BA wherein SB stent malapposition is likely to occur, avoiding the two-stenting and SB treatment with a drug-coated balloon is a considerable treatment.

#### 3.3 SB compromise

The SB compromise after MV stenting is one of the serious complications in bifurcation PCI. It makes guidewire advancement and repairing of the SB vessel structure difficult and leads to procedural myocardial infarction once the SB coronary flow is lost. A large-scale registry study demonstrated that SB occlusion after bifurcation PCI was associated with higher MACE than those without SB occlusion [64]. The SB compromise is also strongly associated with BA; however, the following contradictory opinions have been presented.

*3*.*3*.*1 A narrow BA is related to carina shift*. Vassilev et al. demonstrated that the carina angle between the central lines of the proximal MV and the SB (BA-α, complementary angle of Angle A), was narrowed due to MV straightening after the stenting and that was likely to lead to carina displacement toward the SB with SB narrowing [16]. In their clinical study of 92 bifurcation lesions, SB functional closure was observed in 17.4%, with carina angle (BA-α) <40° as an independent predictor [17].

In the CCTA study of 80 patients with LMCA crossover stenting, LMCA-LAD angle (Angle C) was significantly wider in the SB compromise group than in the non-SB compromise group (140.2±10.3° vs. 132.6±14.2°) [65].

Suárez de Lezo et al. reported that narrower angiographic Angle B was associated with the presence of the "eyebrow" sign (62±23° vs. 76±24°), which was a powerful predictor of SB compromise after MV stenting in non-true bifurcation lesions [66].

In the IVUS study in LMCA-LAD crossover stenting, narrower Angle B was associated with a greater narrowing of the lumen (r = 0.472) and external elastic lamina (r = 0.402) in the LCX after stenting, which suggests that the main mechanism of the LCX ostial compromise is carina shift [67].

In the OCT study, the Angle B between distal MV and SB was not related to SB compromise; however, the carina tip angle ≤ 51° and the length between the branching point and carina tip ≤ 1.75mm were independent predictors for SB compromise [36]. The long and narrow shape of the carina tip has more impact on carina shift after MV stenting than the narrower BA between the daughter vessels.

*3*.*3*.*2 A wide BA is related to SB occlusion*. Dou et al. [24] investigated the risk factors for SB occlusion in 1,601 bifurcation lesions of 1,545 consecutive patients undergoing simple MV stenting in a single center. SB occlusion occurred in 7.4% and multivariate analysis identified higher Angle B as an independent risk factor of SB occlusion. They weighed the severity of the risk of each variable and proposed the RESOLVE score for predicting SB occlusion after MV stenting. In terms of Angle B, <70°, 70°-90°, and ≥90° were scored as 0, 4, and 6 points, respectively. They suggested some explanations for more frequent SB occlusion in the bifurcation with high Angle B. A wide Angle B might promote pressure drop and flow resistance in the SB after MV stenting. The wide Angle B may cause more plaque formation in the bifurcation core which might lead to more plaque shift. Inadequate jailed wire placement due to difficulty in the advancement of guidewire might be related to lesser protection of the SB.

*3*.*3*.*3 Insignificant impact on SB compromise*. Iannaccone et al. investigated SB compromise after provisional stenting in Medina 1-1-1 bifurcation lesion model using computer simulation. SB compromise was most evident in the presence of calcifications, which had a higher impact than BA on SB ostium shape [68].

Such disparity in observations and opinions across studies causes a dilemma regarding the association of the BA to SB compromise. However, it is important to note that the definitions of the BA used in the above studies were quite different. Vassilev et al. [16,17] reported the impact of a narrow BA-α (<40°) on carina shift, but Dou et al. [24] did so for a wide Angle B (>70°) on plaque shift. Therefore, these reports focused on the respective impacts of different angles on SB occlusion and the results might not be mutually exclusive. Further clinical studies are warranted to clarify the impact of these two angles simultaneously. When these special entities for SB compromise are met, aggressive preventive procedure, such as jailed balloon technique [69,70], pre-dilation with kissing balloon inflation [71], and two-stent treatment with upfront SB stenting [72], should be considered.

## Conclusion

The BA defines the geometry of bifurcated vessels and is directly related to coronary shear stress and pathogenesis of atherosclerosis at the bifurcation. 3D measurements in QCA and CCTA are more favorable than 2D QCA for the accurate assessment of the BA. A wider BA is likely to generate more stent malapposition, fracture due to hinge motion, and flow disturbance in the SB ostium, which may be related to frequent clinical events. Carina shift is likely to occur in a bifurcation with a narrower angle between central lines of proximal MV and SB; however, SB occlusion is seen in a bifurcation with a wider angle between distal MV and SB. Although the contradictory results seem to be controversial, the studies were based on different angles. Therefore, comparative studies are warranted to clarify the respective impacts of proximal and distal BA. It is still necessary to predict the impact of the BA on stent configuration, apposition, and SB compromise before bifurcation stenting, which would lead to the optimal selection of stent type and stenting technique.

## Supporting information

S1 Checklist (DOCX)
