# Supplementary material for: Impact of coronary bifurcation angle on the pathogenesis of atherosclerosis and clinical outcome of coronary bifurcation intervention–A scoping review
Source: PLoS One. 2022 Aug 17;17(8):e0273157. doi: 10.1371/journal.pone.0273157 (PMC9385039; doi:10.1371/journal.pone.0273157)
Supplement: S1 Checklist — (DOCX) [file pone.0273157.s001.docx]

| **Section and Topic** | **Item #** | **Checklist item** | **Location where item is reported** |
| --- | --- | --- | --- |
| **TITLE** | | |  |
| Title | 1 | Identify the report as a systematic review. | P1 **Scoping review** |
| **ABSTRACT** | | |  |
| Abstract | 2 | See the PRISMA 2020 for Abstracts checklist. | P2 |
| **INTRODUCTION** | | |  |
| Rationale | 3 | Describe the rationale for the review in the context of existing knowledge. | P3, line 42 -48 |
| Objectives | 4 | Provide an explicit statement of the objective(s) or question(s) the review addresses. | P3, line 49-50 |
| **METHODS** | | |  |
| Eligibility criteria | 5 | Specify the inclusion and exclusion criteria for the review and how studies were grouped for the syntheses. | P3, line 58 - P4, line 68 |
| Information sources | 6 | Specify all databases, registers, websites, organisations, reference lists and other sources searched or consulted to identify studies. Specify the date when each source was last searched or consulted. | P3, line 53-54 |
| Search strategy | 7 | Present the full search strategies for all databases, registers and websites, including any filters and limits used. | P3, line 58 - P4, line 68 |
| Selection process | 8 | Specify the methods used to decide whether a study met the inclusion criteria of the review, including how many reviewers screened each record and each report retrieved, whether they worked independently, and if applicable, details of automation tools used in the process. | P3, line 58 - P4, line 68 |
| Data collection process | 9 | Specify the methods used to collect data from reports, including how many reviewers collected data from each report, whether they worked independently, any processes for obtaining or confirming data from study investigators, and if applicable, details of automation tools used in the process. | P3, line 58 - P4, line 68 |
| Data items | 10a | List and define all outcomes for which data were sought. Specify whether all results that were compatible with each outcome domain in each study were sought (e.g. for all measures, time points, analyses), and if not, the methods used to decide which results to collect. | P3, line 58 - P4, line 68 |
|  | 10b | List and define all other variables for which data were sought (e.g. participant and intervention characteristics, funding sources). Describe any assumptions made about any missing or unclear information. | P3, line 58 - P4, line 68 |
| Study risk of bias assessment | 11 | Specify the methods used to assess risk of bias in the included studies, including details of the tool(s) used, how many reviewers assessed each study and whether they worked independently, and if applicable, details of automation tools used in the process. | P3, line 58 - P4, line 68 |
| Effect measures | 12 | Specify for each outcome the effect measure(s) (e.g. risk ratio, mean difference) used in the synthesis or presentation of results. | P3, line 58 - P4, line 68 |
| Synthesis methods | 13a | Describe the processes used to decide which studies were eligible for each synthesis (e.g. tabulating the study intervention characteristics and comparing against the planned groups for each synthesis (item #5)). | P3, line 58 - P4, line 68 |
|  | 13b | Describe any methods required to prepare the data for presentation or synthesis, such as handling of missing summary statistics, or data conversions. | P3, line 58 - P4, line 68 |
|  | 13c | Describe any methods used to tabulate or visually display results of individual studies and syntheses. | P3, line 58 - P4, line 68 Figure 1 |
|  | 13d | Describe any methods used to synthesize results and provide a rationale for the choice(s). If meta-analysis was performed, describe the model(s), method(s) to identify the presence and extent of statistical heterogeneity, and software package(s) used. | P3, line 58 - P4, line 68 |
|  | 13e | Describe any methods used to explore possible causes of heterogeneity among study results (e.g. subgroup analysis, meta-regression). | Not performed due to scoping review |
|  | 13f | Describe any sensitivity analyses conducted to assess robustness of the synthesized results. | Not performed due to scoping review |
| Reporting bias assessment | 14 | Describe any methods used to assess risk of bias due to missing results in a synthesis (arising from reporting biases). | Not performed due to scoping review |
| Certainty assessment | 15 | Describe any methods used to assess certainty (or confidence) in the body of evidence for an outcome. | Not performed due to scoping review |
| **RESULTS** | | |  |
| Study selection | 16a | Describe the results of the search and selection process, from the number of records identified in the search to the number of studies included in the review, ideally using a flow diagram. | P3, line 58 - P4, line 68  Figure 1 |
|  | 16b | Cite studies that might appear to meet the inclusion criteria, but which were excluded, and explain why they were excluded. | P3, line 58 - P4, line 68  Figure 1 |
| Study characteristics | 17 | Cite each included study and present its characteristics. | P3, line 58 - P4, line 68  Figure 1 |
| Risk of bias in studies | 18 | Present assessments of risk of bias for each included study. | Not performed due to scoping review |
| Results of individual studies | 19 | For all outcomes, present, for each study: (a) summary statistics for each group (where appropriate) and (b) an effect estimate and its precision (e.g. confidence/credible interval), ideally using structured tables or plots. | P4, line 70 – P18, line 341 Tables 1, 2 |
| Results of syntheses | 20a | For each synthesis, briefly summarise the characteristics and risk of bias among contributing studies. | Not performed due to scoping review |
|  | 20b | Present results of all statistical syntheses conducted. If meta-analysis was done, present for each the summary estimate and its precision (e.g. confidence/credible interval) and measures of statistical heterogeneity. If comparing groups, describe the direction of the effect. | Not performed due to scoping review |
|  | 20c | Present results of all investigations of possible causes of heterogeneity among study results. | P4, line 70 – P18, line 341 Tables 1, 2 |
|  | 20d | Present results of all sensitivity analyses conducted to assess the robustness of the synthesized results. | Not performed due to scoping review |
| Reporting biases | 21 | Present assessments of risk of bias due to missing results (arising from reporting biases) for each synthesis assessed. | Not performed due to scoping review |
| Certainty of evidence | 22 | Present assessments of certainty (or confidence) in the body of evidence for each outcome assessed. | P4, line 70 – P18, line 341 |
| **DISCUSSION** | | |  |
| Discussion | 23a | Provide a general interpretation of the results in the context of other evidence. | P4, line 70 – P18, line 341 |
|  | 23b | Discuss any limitations of the evidence included in the review. | P4, line 70 – P18, line 341 |
|  | 23c | Discuss any limitations of the review processes used. | P4, line 70 – P18, line 341 |
|  | 23d | Discuss implications of the results for practice, policy, and future research. | P4, line 70 – P18, line 341 |
| **OTHER INFORMATION** | | |  |
| Registration and protocol | 24a | Provide registration information for the review, including register name and registration number, or state that the review was not registered. | P4, line 70 |
|  | 24b | Indicate where the review protocol can be accessed, or state that a protocol was not prepared. | P3, line 58 - P4, line 68  Figure 1 |
|  | 24c | Describe and explain any amendments to information provided at registration or in the protocol. | P3, line 58 - P4, line 68 |
| Support | 25 | Describe sources of financial or non-financial support for the review, and the role of the funders or sponsors in the review. | P19, line 356-357 |
| Competing interests | 26 | Declare any competing interests of review authors. | P1, line 14 |
| Availability of data, code and other materials | 27 | Report which of the following are publicly available and where they can be found: template data collection forms; data extracted from included studies; data used for all analyses; analytic code; any other materials used in the review. | P19, line 360-362 |

*From:*  Page MJ, McKenzie JE, Bossuyt PM, Boutron I, Hoffmann TC, Mulrow CD, et al. The PRISMA 2020 statement: an updated guideline for reporting systematic reviews. BMJ 2021;372:n71. doi: 10.1136/bmj.n71

For more information, visit: <http://www.prisma-statement.org/>

This is a scoping review which is unlike a systematic review of the effectiveness of a particular intervention based on a precise set of outcomes. Generally, scoping reviews can be used to map the key concepts underpinning a research area as well as to clarify working definitions, and/or the conceptual boundaries of a topic prior to the conduct of a systematic review. Since the impact of bifurcation angle on clinical outcome of coronary bifurcation intervention has been still confused due to the differences in the definition of bifurcation angle and its assessment method, results have been varied. Therefore. we conducted a scoping review in this area. The review was analyzed according to the Joanna Briggs Institute Methodology for JBI Scoping Reviews (reference 12) as in the following checklist of scoping review frame works.

**Scoping review frameworks**

|  | Arksey and O’Malley framework | Enhancements proposed by Levac, Colquhoun and O’Brien. | Location where item is reported |
| --- | --- | --- | --- |
| 1 | Identifying the research question | Clarifying and linking the purpose and research question | P3, line 42-50 |
| 2 | Identifying relevant studies | Balancing feasibility with breadth and comprehensiveness of the scoping process | P3, line 58 - P4, line 68 |
| 3 | Study selection | Using an iterative team approach to selecting studies and extracting data | P3, line 58 - P4, line 68 |
| 4 | Charting the data | Incorporating a numerical summary and qualitative thematic analysis | Figure 1 |
| 5 | Collating, summarizing and reporting the results | Identifying the implications of the study findings for policy, practice or research | P4, line 70 – P18, line 341  Tables 1, 2 |
| 6 | Consultation (optional) | Adopting consultation as a required component of scoping study methodology | Consultation was not done. |

From Institute J.B., The Joanna Briggs Institute reviewers’ manual 2015: Methodology for JBI scoping reviews. Joanne Briggs Inst, 2015: p. 1–24.
